# A Resource and Task Scheduling Based Multi-Objective Optimization Model and Algorithms in Elastic Optical Networks

**DOI:** 10.3390/s22249579

**Published:** 2022-12-07

**Authors:** Yuping Wang, Qingdong Yang, Xiaofang Guo

**Affiliations:** 1 School of Science, Xi’an Technological University, Xi’an 710021, China; 2 School of Computer Science and Technology, Xidian University, Xi’an 710071, China

**Keywords:** elastic optical networks, data center allocation, routing, spectrum allocation, virtual network function, multi-objective optimization, evolutionary algorithm

## Abstract

The elastic optical network (EON) adopting virtual network function (VNF) is a new type of network, in which the routing, spectrum, and data center allocation are key and challenging problems, and solving these three problems simultaneously can not only improve the network efficiency for network providers, but also let users obtain better service. However, few existing works handle these three problems simultaneously. To tackle the three problems simultaneously, given a set of network function chains (i.e., a set of tasks), we set up a new multi-objective optimization model in which the total length of paths for all tasks is minimized, the totally occupied spectrums are minimized, and the loads on all data centers are most balanced, simultaneously. To solve the model, we design two new evolutionary algorithms. The experiments are conducted on 16 cases of 4 widely used types of networks, and the results indicate that the proposed model and algorithms are effective.

## 1. Introduction

With the development of internet and network technology, a wavelength division multiplexing (WDM) network can not satisfy the requirement of real world problems due to its spectrum allocation mode of fixed grid and low spectrum utilization. Elastic optical network (EON) is a potential direction for developing the future network due to flexible spectrum allocation and high spectrum utilization. However, the routing and spectrum allocation (RSA) problem in EON is more complex. RSA has become one of the hottest and key problems in EON [1]. It needs to handle multiple issues simultaneously (e.g., select the best route, use the least spectrum resource, make the communication efficient, etc.). Although many researchers have studied these kinds of problems, most of them only can handle one or two issues simultaneously. For example, Ref. [2] proposed an adaptive scheme which only tackles the routing and spectrum allocation problems based on reducing the relative cost of network flow and the probability of communication block. Ref. [3] proposed a RSA strategy which only improves the quality of transmission (QoT) based on both the link state advertisement (LSA) and the risk reduction of quality of transmission (QoT). To improve the communication quality by tackling unbalanced distribution of network flows in EON, Ref. [4] proposed two algorithms based on the OTTM model to improve bandwidth efficiency. To handle routing and spectrum issues, Ref. [5] proposed an adaptive RSA algorithm which tried to select the routing of the lowest relative cost and design the efficient spectrum scheme by estimating both the transient effect of call admission on a given set of routing/spectrum and the relative cost of reaching the forward destination of the calls. To handle the spectrum problem, Ref. [6] set up an integer linear programming model for spectrum allocation problems by analyzing the characteristic of network data flow of three kinds of elastic optical networks, and proposed two meta heuristic algorithms based on particle swarm optimization and tabu search. However, these works did not consider how to tackle more than two issues simultaneously and cannot complete a series of function service chains (FSC).

In addition, with the fast increase of the number of network users, it becomes more difficult to handle multiple issues simultaneously, and it is required to continuously update the network hardware with the increase of tasks and users. However, the hardware update speed can not catch the speed of increase of tasks and users. Therefore, it is impossible to solve this problem by only updating the hardware. The virtual network function (VNF) is to realize the function of special hardware by using software without the need for updating hardware. VNF can increase the number of tasks/users and enhance the network resource utilization without increasing network hardware. Thus, VNF is a potential technique in the environment of fast increase of users/tasks for elastic optical networks. Moreover, the elastic optical network with VNF provides an efficient tool to possibly handle multiple issues simultaneously in the RSA problem and complete a series of function service chains (FSC). Each FSC (can be seen as a task) consists of a sequential functions (e.g., data encryption, data decryption, deep packet inspection, data monitoring, etc.) to be successively completed. However, this flexibility and adaptability will result in more complexity for setting up models and designing algorithms. The existing models and algorithms for elastic optical networks can only handle one or two issues simultaneously, and cannot tackle multiple (more than two) issues simultaneously. In fact, the current models and algorithms for elastic optical networks face many challenges. One of the most difficult challenges is how to tackle the following four problems on EON simultaneously:

(1) How to reasonably make the routing (select reasonable path for each task/FSC) such that data can be quickly transmitted; (2) how to efficiently allocate spectrums such that the spectrum resource is conserved the most; (3) how to make the loads most balanced among the data centers such that the network communication is more efficient and stable; and (4) how to deploy VNF efficiently.

## 2. Related Works

Currently, there have been some research works in this field. For example, in order to handle two problems (i.e., reduce the cost and improve the quality of service (QoS)), Ref. [7] proposed an integrated algorithm based on VNF migration strategy, which aims at reducing the income loss of network service provider due to both data lost during the transmission and decreasing of quality of service (QoS), but it did not consider how to balance the loads among the data centers. To solve VNF and physical function allocation problems in network and cloud environments, Ref. [8] proposed a heuristic algorithm based on a self-defined greedy strategy to improve the performance and ability of feature decomposition method, but it did not consider the spectrum allocation and load balance. Ref. [9] surveyed the current research progress on VNF allocation problems. Ref. [10] proposed a new virtualization method on network functions, but it did not consider the load balance and routing. In order to balance the load of the data center nodes, Ref. [11] proposed a highly efficient switch migration (HESM) method for controlling load balance via minimizing migration cost, but it did not consider the routing and spectrum allocation. To increase spectral efficiency for the routing and spectrum assignment (RSA) in elastic optical networks (EON), Ref. [12] proposed a novel technique for resource planning and consumption estimation by the optimal utilization of already existing resources, but it did not consider the load balance. In order to enhance the efficiency of routing and resource allocation, Ref. [13] proposed a prediction-based dynamic slicing mechanism (PDSM) for heterogeneous elastic fiber wireless networks, but it also did not consider the load balance. To obtain a better resource allocation scheme, Ref. [14] proposed a novel constrained deep reinforcement learning algorithm for the resource allocation problem. Aiming at improving the efficiency of both wireless resource management and computation resource management within network slices in edge computing systems, Ref. [15] proposed an efficient approximation algorithm based on the game theory, which minimizes the completion time of task execution in the system. These algorithms also did not consider the load balance. Ref. [16] investigated static routing, VNF, and spectrum allocation (RMSTA) of advanced reservation (AR) requests in elastic optical networks. They formulated a two-objective optimization model by minimizing the occupied spectrum and time resources, and proposed a heuristic algorithm based on three sorting strategies. However, this study focused only on sorting requests and neglected the effect of routing and spectrum allocation on the model and algorithm. Furthermore, solving the formulated multi-objective problem by minimizing the weighted sum of two objectives cannot obtain a set of solutions, which failed to satisfy users with different preferences. Ref. [17] investigated the routing and content replication placement in elastic optical networks. They built a mixed integer linear programming model and proposed a heuristic optimization method. However, this study focused on content replication and neglected the impact of routing policies on designing algorithms in large-scale networks and short-term re-optimization scenarios. Ref. [18] proposed an ILP model that takes into account the multicast routing, spectrum, and transceivers for multicast requests, with the goal of minimizing the spectrum consumption and transceivers required for the subtree in an elastic optical data center network (EODCNs). However, this study did not give a method to determine the weight coefficients for each optimization objective in the ILP model and did not consider the load balance. Ref. [19] used a physical slicing technique that supports an end-to-end optical path on a combination of multiple discontinuous spectral slots in order to enhance the spectrum allocation, and proposed a scheme to determine the splitting position and required number of slots for each slice component in an EON network. They built a mixed integer linear programming (MILP) model by making the trade-off among the slicers availability, spectrum utilization, and bandwidth blocking probability. Although the physical slicing technology has improved the spectrum utilization rate in EON, this study did not consider the network load balance. Ref. [20] studied the allocation of data center and spectrum for VNF service chain in inter-datacenter elastic optical networks. To minimize the total network cost and balance the utilization of resources, the authors set up a model with three optimization objectives first, and then transformed it into a single-objective optimization model by using the weight sum method. Finally, they proposed a heuristic algorithm to reduce the congestion in both data centers and links. However, this work ignored the communication quality (e.g., quality of service). To deploy VNF effectively, Ref. [21] proposed a deep reinforced learning based algorithm to minimize the spectrum utilization ratio and the number of deployed VNFs jointly. A trade-off between spectrum utilization ratio and number of deployed VNFs was made by assigning a weight to each of the objectives. However, this method did not carefully take into account both routing and load balance of data centers. Instead, it only used a simple heuristic strategy to make the routing.

By summary, the existing works mainly focus on tackling only one or two issues of the four aforementioned issues. There have been few works tackling the four problems above simultaneously. To overcome this shortcoming, the main contributions in this paper are as follows: (1) We set up a new multi-objective model for solving all these problems simultaneously, i.e., select reasonable routes for each task such that data can be quickly transmitted; efficiently allocate spectrums such that the spectrum resource is conserved the most; make the loads most balanced among the data centers such that the network communication is more efficient and stable; and deploy VNF efficiently. (2) We propose two new evolutionary algorithms for solving this model. (3) We conduct a lot of experiments (including 16 cases of 4 types of widely used networks) to test the effectiveness and efficiency of the proposed model and algorithms.

The rest of the paper is arranged as follows: In Section 3, we first briefly introduce the problem considered in this paper, and then set up a new optimization model for the problem. Finally, we design two new evolutionary algorithms for the model. In Section 4, we conduct the experiments and conduct the analysis of experimental results. In Section 5, we make the discussion on the paper and point out the future work. Conclusions are made in Section 6.

## 3. Problem Description, Optimization Model, and Algorithm

### 3.1. Problem Description

A scheduling problem of the elastic optical network using virtual network functions can be described as follows: The elastic optical network using virtual network functions can be represented by an undirected graph G(V,E), where the set $V=\{v_{1},v_{2},\cdots ,v_{N_{v}}\}$ of the vertexes in the graph represents the set of nodes of the network, the set of nodes of data centers is denoted by $V_{D}=\left\{v_{1},v_{2},\cdots ,v_{N_{d}}\right\}$, and the set $E=\{L_{ij}|v_{i},v_{j}\in V\}$ of edges of graphs represents the set of links of networks. The set of tasks is denoted by $\mathrm{FSC}=\{r_{1},r_{2},\cdots ,r_{k},\cdots ,r_{N_{R}}\}$, where each task is a function service chain (FSC), and the *k*-th function service chain (also called the *k*-th task) is represented as $r_{k}=\left(s_{k},d_{k},b_{k},t_{k}\right)$, where $s_{k}$ is source node, $d_{k}$ is the destination node, $t_{k}$ is the function sequence $t_{k}=\left\{t_{k}^{1},t_{k}^{2},\cdots ,t_{k}^{i},\cdots ,t_{k}^{F_{k}}\right\}$ required to be realized sequentially by task $r_{k}$, where $t_{k}^{i}\in \left\{1,2,\cdots ,N_{T}\right\}$ is the index of the *i*-th virtual network function to be realized by the *k*-th task $r_{k}$ (for convenience, the virtual network function is briefly called the function in the following). $t_{k}^{i}$ are different each other in $t_{k}$. $F_{k}$ is the number of the functions contained in the *k*-th FSC. $N_{T}$ is the total number of all different functions in all $N_{R}$ tasks. $b_{k}$ is the number of slots to be occupied by the *k*-th task $r_{k}$. $N_{F}$ is the maximal number of slots which are available in the path.

The problem considered in this paper is to tackle the following issues: (1) how to select a proper path from the source node to the destination node for each task such that the total length of all paths is minimized; (2) how to assign the slots in the selected path for each task such that the slot assumption is minimized; (3) how to make the loads most balanced among the data centers such that the network communication is more efficient and stable; and (4) how to deploy VNF efficiently.

### 3.2. A Multi-Objective Optimization Model

#### 3.2.1. Determining Variables for the Model

A. Path variables:

Let $P_{k}=\left(s_{k},x_{k}^{1},x_{k}^{2},\cdots ,x_{k}^{l_{k}},d_{k}\right)$ denote the path to be chosen for the task $r_{k}$, where $x_{k}^{i}\in \left\{1,2,\cdots ,N_{v}\right\}$, $x_{k}^{1}$ is the first node of the path to reach from the start node $s_{k}$, and $x_{k}^{i}$ is the *i*-th node of the path to reach from the start node $s_{k}$ for $i=1,2,\cdots ,l_{k}$. Finally, node $x_{k}^{l_{k}}$ directly reaches the destination node $d_{k}$. That is, path $P_{k}$ is as follows:

$$s_{k}\to x_{k}^{1}\to x_{k}^{2}\to \cdots \to x_{k}^{l_{k}}\to d_{k}$$

and has to pass through at lest one data center node. For example, path $P_{k}=\left(5,3,6,1,8,7,2\right)$ for task $r_{k}$ means that the source node is node 5; then, the first node to reach from source node 5 is node 3, and the second node to reach from source node 5 is node 6. Similarly, the later nodes in the path are sequentially node 1, node 8, node 7, and finally to the destination node 2.

Let $x_{k}=\left(x_{k}^{1},x_{k}^{2},\cdots ,x_{k}^{l_{k}}\right)$ denote path variables, and let $V_{v_{i}}$ denote the set of nodes connected to node $v_{i}$ in the network. Then, $x_{k}^{1}\in V_{s_{k}},x_{k}^{2}\in V_{x_{k}^{1}},x_{k}^{3}\in V_{x_{k}^{2}},\cdots ,x_{k}^{l_{k}}\in V_{x_{k}^{l_{k}-1}},d_{k}\in V_{x_{k}^{l_{k}}}$, and $D_{c}\cap \left\{s_{k},x_{k}^{1},x_{k}^{2},\cdots ,x_{k}^{l_{k}},d_{k}\right\}\neq \emptyset ,$ where $D_{c}$ is the set of nodes of data centers.

In the following, we shall design the following Algorithm 1 to look for such a path

$$P_{k}=\left(s_{k},x_{k}^{1},\cdots ,x_{k}^{l_{k}},d_{k}\right)$$

in which at least one node is a data center node, and to determine the path variables.    
**Algorithm 1** Path search algorithm**Step 1:** Select $x_{k}^{1}$. For $s_{k}$, if $V_{s_{k}}$ contains any data center node, randomly select one data center node as $x_{k}^{1}$; otherwise, randomly select a node in $V_{s_{k}}$ as $x_{k}^{1}$, and let i=1. 
**Step 2:** Select $x_{k}^{i+1}$ in $V_{k}^{i}/\left\{s_{k},x_{k}^{1},\cdots ,x_{k}^{i}\right\}$ according to the following two cases, where $V_{k}^{i}$ represents $V_{x_{k}^{i}}$ for notation convenience:

Case I: $\left\{s_{k},x_{k}^{1},\cdots ,x_{k}^{i}\right\}$ contains a data center node. If $d_{k}\in V_{k}^{i}$, select $d_{k}$ as $x_{k}^{i+1}$; otherwise, randomly select a node in $V_{k}^{i}$ as $x_{k}^{i+1}$.

Case II: $\left\{s_{k},x_{k}^{1},\cdots ,x_{k}^{i}\right\}$ does not contain any data center node. If $V_{k}^{i}$ contains any data center node, randomly select one data center node in $V_{k}^{i}$ as $x_{k}^{i+1}$, let i=i+1, go to step 2; otherwise, randomly select one node in $V_{k}^{i}$ as $x_{k}^{i+1}$, go to step 3. 
**Step 3:** If $x_{k}^{i+1}\neq d_{k}$, let i=i+1, go to step 2 (the current path neither contains any data center node nor reaches $d_{k}$), otherwise, go to step 4 (the current path does not contain any data center node but reaches $d_{k}$. This is not a feasible path, and we have to select another path). 
**Step 4:** Re-select the nodes after node $x_{k}^{i}$ until selecting node $d_{k}$ by using the previous steps. If the new path is still an infeasible path (containing no data center node), re-select nodes after $x_{k}^{i-1}$ until selecting $d_{k}$. Repeat this process until one feasible path is found.


B. Slot variables:

Let $y_{k}=\left(y_{k}^{1},y_{k}^{2},\cdots ,y_{k}^{N_{F}}\right)$ denote the slot assignment on path $P_{k}$ for task $r_{k}$, where each $y_{k}^{i}$ is a boolean variable. $y_{k}^{i}=1$ represents that task $r_{k}$ uses the *i*-th slot of each edge of path $P_{k}$, and $y_{k}^{i}=0$ means that task $r_{k}$ does not use the *i*-th slot on path $P_{k}$. Let $U_{k}$ represent the set of tasks whose paths share a common link with path $y_{k}$, i.e.,

$$U_{k}=\left\{v\;|\;P_{k}\cap P_{v}\neq \emptyset ,\;\forall 1\leq v\leq N_{R}\right\}.$$

$y_{k}$ must satisfy the following conditions: (1) The number of slots used in slot assignment $y_{k}$ should be equal to the number of slots required by task $r_{k}$; (2) For the same slot, it should be used by at most one task. That is,

(1) $$\left\{\begin{array}{c} \sum_{i=1}^{N_{F}}y_{k}^{i}=b_{k},k=1,2,\cdots ,N_{R}\\ \sum_{v\in U_{k}}y_{v}^{i}\leq 1,i=1,2,\cdots ,N_{F} \end{array}\right.$$

C. Function variables:

Suppose the data center nodes passed successively through by $P_{k}$ are:

$$V_{k_{1}},V_{k_{2}},\cdots ,V_{k_{i_{k}}}$$

The numbers of functions in $r_{k}$ assigned to data center nodes $V_{k_{1}},V_{k_{2}},\cdots ,V_{k_{i_{k}}}$ on path $P_{k}$ are

$$z_{k}=\left(z_{k}^{1},z_{k}^{2},\cdots ,z_{k}^{i_{k}}\right),$$

respectively, where $z_{k}^{j}$ is the number of functions assigned to data center $V_{k_{j}}$ to be realized, and the sequence of these functions assigned to the data center nodes is the same as that in $r_{k}$. We call $z_{k}$ the function variables, which should satisfy the following conditions: The total number of assigned functions should be equal to the number of functions required to be realized in $r_{k}$, i.e.,

$$\sum_{j=1}^{i_{k}}z_{k}^{j}=F_{k},k=1,\cdots ,N_{R}$$

where $F_{k}$ is the number of functions required to be realized by $r_{k}$.

#### 3.2.2. Objective Function Determination

A. Minimize the total length of all paths:

Note that path $P_{k}$ corresponding to task $r_{k}$ has $\left(l_{k}+1\right)$ edges, and its length is $\left(l_{k}+1\right)$. Then, the total length of all paths is $\sum_{k=1}^{N_{R}}\left(l_{k}+1\right)$.

Thus, the first objective is to minimize the following total length:

$$f_{1}\left(x_{1},\cdots ,x_{N_{R}}\right)=\sum_{k=1}^{N_{R}}\left(l_{k}+1\right)$$

Denote $x=(x_{1},\cdots ,x_{N_{R}})$. Then, we have $f_{1}\left(x\right)=\sum_{k=1}^{N_{R}}\left(l_{k}+1\right)$.

B. Minimize the total number of slots used:

The number of slots in each link of path $P_{k}$ for task $r_{k}$ is $b_{k}$, and the number of links of path $P_{k}$ is $l_{k}^{{}^{\prime }}$ (excluding the shared common links and the shared links are not computed repeatedly). Thus, the total number of slots used by all tasks is

$$f_{2}\left(x,y_{1},y_{2},\cdots ,y_{N_{R}}\right)=\sum_{k=1}^{N_{R}}b_{k}*l_{k}^{{}^{\prime }}$$

Denote $y=(y_{1},\cdots ,y_{N_{R}})$. Then, the second objective is to minimize the following function $f_{2}\left(x,y\right)=\sum_{k=1}^{N_{R}}b_{k}*l_{k}^{{}^{\prime }}$.

C. Optimize the balance of loads on all data center nodes:

To optimize the balance of loads on all data center nodes, the number of functions of all $N_{R}$ tasks should be assigned to these data center nodes as equally as possible. The index set of the data center nodes passed through by path $P_{k}$ is denoted by $\left\{k_{1},k_{2},\cdots ,k_{i_{k}}\right\}$. Let

$$\tilde{D_{c}}=\overset{N_{R}}{\underset{k=1}{⋃}}\left\{k_{1},k_{2},\cdots ,k_{i_{k}}\right\}$$

denote the index set of data center nodes used by all tasks.

For $\forall c\in \tilde{D_{c}}$, the number of functions handled by data center node *c* is

$$q_{c}\left(z_{1},\cdots ,z_{N_{R}}\right)=\sum_{k\in \tilde{D_{c}}}z_{k}^{c}$$

The standard deviation of the numbers of functions handled by all data center nodes is

$$f_{3}\left(x,y,z_{1},z_{2},\cdots ,z_{N_{R}}\right)=\sqrt{\frac{1}{|\tilde{D_{c}}|}\sum_{c\in \tilde{D_{c}}}{\left[q_{c}\left(z_{1},\cdots ,z_{N_{R}}\right)-\frac{N_{T}}{|\tilde{D_{c}}|}\right]}^{2}}$$

Denote $z=(z_{1},\cdots ,z_{N_{R}})$. Then, optimizing the balance of load of data centers is equal to minimizing the following objective:

$$f_{3}\left(x,y,z\right)=\sqrt{\frac{1}{|\tilde{D_{c}}|}\sum_{c\in \tilde{D_{c}}}{\left[q_{c}\left(z\right)-\frac{N_{T}}{|\tilde{D_{c}}|}\right]}^{2}}$$

Now our goal is to minimize the above three objectives simultaneously, i.e.,

$$\min\left\{f_{1}\left(x\right),f_{2}\left(x,y\right),f_{3}\left(x,y,z\right)\right\}$$

#### 3.2.3. Constraints

A. The path variable constraints:

Path $P_{k}$ selected for task $r_{k}$ should pass through at least one data center node, i.e.,

$$D_{c}⋂\{s_{k},x_{k}^{1},x_{k}^{2},\cdots ,x_{k}^{l_{k}},d_{k}\}\neq \emptyset ,k=1,\cdots ,N_{R}$$

B. Slot constraints:

$$\sum_{i=1}^{N_{F}}y_{k}^{i}=b_{k},k=1,\cdots ,N_{R}$$

$$\sum_{v\in U_{k}}y_{v}^{i}\leq 1,i=1,2,\cdots ,N_{F}$$

C. Function assignment constraints:

$$\sum_{j=1}^{i_{k}}z_{k}^{j}=F_{k},k=1,\cdots ,N_{R}$$

#### 3.2.4. Multi-Objective Optimization Model

By summary, we can set up the following multi-objective optimization model:

(2) $$\left\{\begin{array}{c} \min\left\{f_{1}\left(x\right),f_{2}\left(x,y\right),f_{3}\left(x,y,z\right)\right\}\\ s.t.\\ \left(1\right)D_{c}⋂\{s_{k},x_{k}^{1},x_{k}^{2},\cdots ,x_{k}^{l_{k}},d_{k}\}\neq \emptyset ,k=1,\cdots ,N_{R}\\ \left(2\right)\sum_{i=1}^{N_{F}}y_{k}^{i}=b_{k},k=1,\cdots ,N_{R}\\ \left(3\right)\sum_{v\in U_{k}}y_{v}^{i}\leq 1,i=1,2,\cdots ,N_{F}\\ \left(4\right)\sum_{j=1}^{i_{k}}z_{k}^{j}=F_{k},k=1,\cdots ,N_{R} \end{array}\right.$$

### 3.3. Two Evolutionary Algorithms for Solving the Model

Note that the above model is a nonlinear integer multi-objective optimization model. There is no existing algorithm for this model. We shall design two evolutionary algorithms to solve this model in this section.

The framework of the first algorithm is as follows: First, design a strategy to generate the initial population. Second, design a mutation operator. Finally, evolve the population iteratively and obtain a set of the approximate solutions. In the following, we shall introduce each part of the algorithm, respectively.

#### 3.3.1. A Strategy for Generating the Initial Population

Given the population size $N_{pop}$, any individual (x,y,z) in the initial population $POP^{0}$ is generated by the following Algorithm 2:
**Algorithm 2** A strategy for generating the initial population**Step 1:** Generate each *x* by using Algorithm 1. **Step 2:** Generate each $y={\left(y_{1},y_{2},\cdots ,y_{N_{R}}\right)}^{T}$ as follows: denote
$$y=\left(\begin{array}{c} y_{1}\\ y_{2}\\ \cdots \\ y_{N_{R}} \end{array}\right)=\left(\begin{array}{cccc} y_{1}^{1} & y_{1}^{2} & \cdots & y_{1}^{N_{F}}\\ y_{2}^{1} & y_{2}^{2} & \cdots & y_{2}^{N_{F}}\\ \cdots & \cdots & \cdots & \cdots \\ y_{N_{R}}^{1} & y_{N_{R}}^{2} & \cdots & y_{N_{R}}^{N_{F}} \end{array}\right)$$
Set initial matrix y=0. Let $y_{w_{1}},y_{w_{2}}\cdots ,y_{w_{N_{R}}}$ be a random sequence of $y_{1},y_{2},\cdots ,y_{N_{R}}$ and $y_{w}$ denote the matrix whose rows are $y_{w_{1}},y_{w_{2}}\cdots ,y_{w_{N_{R}}}$, respectively. Set $y_{w_{1}}^{1}=y_{w_{1}}^{2}=\cdots =y_{w_{1}}^{b_{w_{1}}}=1$, and $y_{w_{1}}^{j}=0$ for $j\neq 1,2,\cdots ,b_{w_{1}}$. If path $P_{w_{2}}$ for task $r_{w_{2}}$ shares a link with path $P_{w_{1}}$ for task $r_{w_{1}}$, set $y_{w_{2}}^{j}=1$ for $b_{w_{1}}+1\leq j\leq b_{w_{1}}+b_{w_{2}}$ and $y_{w_{2}}^{j}=0$ for other *j*. Otherwise, set $y_{w_{2}}^{1}=y_{w_{2}}^{2}=\cdots =y_{w_{2}}^{b_{w_{2}}}=1$, and $y_{w_{2}}^{j}=0$ for $j\neq 1,2,\cdots ,b_{w_{2}}$. Generally, for each $k\in \{2,3,\cdots ,N_{R}\}$, if path $P_{w_{k}}$ for task $r_{w_{k}}$ does not share a link with any previous path $P_{w_{j}}$ for 1≤j≤k−1, set $y_{w_{k}}^{1}=y_{w_{k}}^{2}=\cdots =y_{w_{k}}^{b_{w_{k}}}=1$, and $y_{w_{k}}^{j}=0$ for $j\neq 1,2,\cdots ,b_{w_{k}}$. Otherwise, let $U_{w_{k}}$ denote the set of tasks whose paths share a link with path $P_{w_{k}}$ for task $w_{k}$. Compute
$${\bar{y}}_{w_{k}}=\sum_{v\in U_{w_{k}}}y_{v}$$Let
$$k_{0}=\min\left\{k_{0}^{\prime }|\;\sum_{i=k_{0}^{\prime }}^{k_{0}^{\prime }+b_{w_{k}}-1}{\bar{y}}_{w_{k}}^{i}=0\;for\;k_{0}^{\prime }\in \left\{1,2,\cdots ,N_{F}-b_{w_{k}}\right\}\right\}$$
Assign the slots $k_{0},k_{0}+1,\cdots ,k_{0}+b_{w_{k}}-1$ to path $P_{w_{k}}$ for task $r_{w_{k}}$, i.e, let $y_{w_{k}}^{k_{0}}=y_{w_{k}}^{k_{0}+1}=\cdots =y^{k_{0}+b_{w_{k}}-1}=1$, and $y_{w_{k}}^{j}=0$ for $j\neq k_{0},k_{0}+1,\cdots ,k_{0}+b_{w_{k}}-1$.
Then, *y* satisfies the constraints:$\sum_{i=1}^{N_{F}}y_{k}^{i}=b_{k},k=1,\cdots ,N_{R}$
$$\sum_{v\in U_{k}}y_{v}^{i}\leq 1,i=1,2,\cdots ,N_{F}$$**Step 3:** Suppose the data center nodes passed successively through by path $P_{k}$ are:$$V_{k_{1}},V_{k_{2}},\cdots ,V_{k_{i_{k}}}$$
The numbers of functions in task $r_{k}$ assigned to data center nodes $V_{k_{1}},V_{k_{2}},\cdots ,V_{k_{i_{k}}}$ are denoted by $z_{k}^{1},z_{k}^{2},\cdots ,z_{k}^{i_{k}}$, respectively, where $z_{k}^{j}$ is the number of functions assigned to data center $V_{k_{j}}$ for $j=1,2,\cdots ,i_{k}$. Denote
$$z_{k}=\left(z_{k}^{1},z_{k}^{2},\cdots ,z_{k}^{i_{k}}\right)$$
For each task $r_{k}$, we can generate $z_{k}$ by randomly taking $i_{k}$ non-negative integers $z_{k}^{1},z_{k}^{2},\cdots ,z_{k}^{i_{k}}\in \left[0,F_{k}\right]$ such that $\sum_{j=1}^{i_{k}}z_{k}^{j}=F_{k},k=1,\cdots ,N_{R}$.**Step 4:** Repeat Step 1 to Step 3 for $N_{pop}$ times to obtain $POP^{0}$.


#### 3.3.2. Mutation Operator

For any individual (x,y,z) in the current population $POP^{t}$, the mutation operator for it is as follows:

(1) Scheme of mutation for *x*:

1. For each $x_{k}$, generate ${\bar{x}}_{k}^{1}$. For $s_{k}$, randomly select a node in $V_{s_{k}}/x_{k}^{1}$ as ${\bar{x}}_{k}^{1}$.

2. Generate ${\bar{x}}_{k}^{i+1}$ in $V_{k}^{i}/\left\{s_{k},{\bar{x}}_{k}^{1},\cdots ,{\bar{x}}_{k}^{i}\right\}$ according to the following two cases:

Case I: $\left\{s_{k},{\bar{x}}_{k}^{1},\cdots ,{\bar{x}}_{k}^{i}\right\}$ contains a data center node. If

$$\left\{\left\{x_{k}^{1},x_{k}^{2},\cdots ,d_{k}\right\}-\left\{{\bar{x}}_{k}^{1},\cdots ,{\bar{x}}_{k}^{i}\right\}\right\}\cap V_{k}^{i}\neq \emptyset ,$$

select one node

$$x_{k}^{i^{{}^{\prime }}}\in \left\{\left\{x_{k}^{1},x_{k}^{2},\cdots ,d_{k}\right\}-\left\{{\bar{x}}_{k}^{1},\cdots ,{\bar{x}}_{k}^{i}\right\}\right\}\cap V_{k}^{i},$$

and let ${\bar{x}}_{k}=\left(s_{k},{\bar{x}}_{k}^{1},\cdots ,{\bar{x}}_{k}^{i},x_{k}^{i^{{}^{\prime }}},\cdots ,d_{k}\right)$. We obtain the mutation offspring ${\bar{x}}_{k}$ of $x_{k}$; otherwise, randomly select a node in $V_{k}^{i}$ as ${\bar{x}}_{k}^{i+1}$. Go to step 3.

Case II: $\left\{s_{k},{\bar{x}}_{k}^{1},\cdots ,{\bar{x}}_{k}^{i}\right\}$ does not contain any data center node. If $V_{k}^{i}$ contains any data center node, randomly select one data center node in $V_{k}^{i}$ as ${\bar{x}}_{k}^{i+1}$. Let i=i+1, go to step 2; otherwise, randomly select one node in $V_{k}^{i}$ as ${\bar{x}}_{k}^{i+1}$, and go to step 3.

3. If ${\bar{x}}_{k}^{i+1}\notin \left\{x_{k}^{1},x_{k}^{2},\cdots ,d_{k}\right\}$, let i=i+1, and go to step 2; otherwise, go to step 4.

4. Select

$$i^{{}^{\prime }}\in \left\{v|x_{k}^{v}={\bar{x}}_{k}^{i+1},x_{k}^{v}\in \left\{x_{k}^{1},x_{k}^{2},\cdots ,d_{k}\right\}\right\},$$

and let ${\bar{x}}_{k}=\left(s_{k},{\bar{x}}_{k}^{1},\cdots ,{\bar{x}}_{k}^{i+1},x_{k}^{i^{{}^{\prime }}+1},\cdots ,d_{k}\right)$. If ${\bar{x}}_{k}$ does not contain any data center node, go to step 5; otherwise, we obtain the mutation offspring ${\bar{x}}_{k}$ of $x_{k}$.

5. Re-select the nodes after node ${\bar{x}}_{k}^{i}$ until selecting node $d_{k}$ by using the previous steps. If the new path is still an infeasible path (containing no data center node), re-select nodes after $x_{k}^{i-1}$ until selecting $d_{k}$. Repeat this process until one feasible path is found.

(2) Scheme of mutation for *y*: Randomly change two elements of $y_{w_{1}},y_{w_{2}}\cdots ,y_{w_{N_{R}}}$, and obtain the mutation offspring $\bar{y}$ of *y* by step 2 of Algorithm 2.

(3) Scheme of mutation for *z*: Generate the mutation offspring $\bar{z}$ of *z* by step 3 of Algorithm 2.

Note that the offspring $\left(\bar{x},\bar{y},\bar{z}\right)$ of (x,y,z) generated by the above schemes satisfies all constraints of the model. Let $O^{t}$ denote the set of offspring of $POP^{t}$.

#### 3.3.3. Selection Operator

We select the next generation population $POP^{t+1}$ among $POP^{t}\cup O^{t}$ by using the selection operator in NSGA-II [22].

#### 3.3.4. The First Evolutionary Algorithm for the Model

The first proposed algorithm is the following Algorithm 3:
**Algorithm 3 An evolutionary algorithm for the model****Step 1:** Given the maximum generation number $N_{gen}$ and population size $N_{pop}$. 
**Step 2:** Generate initial population $POP^{0}$ by using Algorithm 2, and set t=0. **Step 3:** Mutation. For each individual (x,y,z) in the current population $POP^{t}$, execute the mutation operator on it to generate its offspring $(\bar{x}$, $\bar{y}$, $\bar{z})$. All offspring $\left(\bar{x},\bar{y},\bar{z}\right)$ form an offspring population $O^{t}$. **Step 4:** Selection. Select the best $N_{pop}$ individuals among $POP^{t}\cup O^{t}$ to form the next generation population $POP^{t+1}$ by selection operator. Set t=t+1. **Step 5:** If $t\geq N_{gen}$, the non-dominated set of $POP^{t}$ is the approximate Pareto optimal solution set. Stop. Otherwise, go to Step 2.


#### 3.3.5. The Second Evolutionary Algorithm for the Model

The second proposed algorithm is the following Algorithm 4:
**Algorithm 4 Another evolutionary algorithm for the model**The steps of Algorithm 4 are the same as those of Algorithm 3 except for the replacement of Algorithm 1 in Algorithm 3 by the following Algorithm 5 to generate path variable  

The proposed algorithm for generating path variavles in Algorithm 4 is as follows:
**Algorithm 5 Strategy for generating path variable *x*****Step 1:** For each task $r_{k}$, randomly select $i_{k}$ data center nodes, and their random sequence is denoted by $D_{k}=k_{1}^{{}^{\prime }},k_{2}^{{}^{\prime }},\cdots ,k_{i_{k}}^{{}^{\prime }}$.  **Step 2:** Use the Dijkstra algorithm [23] to obtain the shortest path from $s_{k}$ to $k_{1}^{{}^{\prime }}$, recorded as $Path1:s_{k}\to \cdots \to k_{1}^{{}^{\prime }}$.  **Step 3:** Use the Dijkstra algorithm to obtain the shortest path from $k_{i_{k}}^{{}^{\prime }}$ to $d_{k}$, recorded as $Path3:k_{i_{k}}^{{}^{\prime }}\to \cdots \to d_{k}$.  **Step 4:** Use the Dijkstra algorithm to obtain the shortest path between each pair of two adjacent data centers $k_{i}^{{}^{\prime }}$ and $k_{i+1}^{{}^{\prime }}$ for $i=1,2,\cdots ,i_{k}-1$. This path is denoted by $p_{\left(k_{i}^{{}^{\prime }},k_{i+1}^{{}^{\prime }}\right)}$ for $i=1,2,\cdots ,i_{k-1}$. Connect these paths successively to obtain a path through data center node sequence $D_{k}=k_{1}^{{}^{\prime }},k_{2}^{{}^{\prime }},\cdots ,k_{i_{k}}^{{}^{\prime }}$:$$Path2=\{p_{\left(k_{1}^{{}^{\prime }},k_{2}^{{}^{\prime }}\right)}\to p_{\left(k_{2}^{{}^{\prime }},k_{3}^{{}^{\prime }}\right)},\cdots ,\to p_{\left(k_{i}^{{}^{\prime }},k_{i+1}^{{}^{\prime }}\right)},\cdots ,\to p_{\left(k_{i_{k}-1}^{{}^{\prime }},k_{i_{k}}^{{}^{\prime }}\right)}\}$$**Step 5:** Connect the tail node of Path1 with the head node of Path2, connect the tail node of Path2 with the head node of Path3, and then obtain the path {Path1→Path2→Path3} of task $r_{k}$. Denote this path by $\left(s_{k},x_{k}^{1},x_{k}^{2},\cdots ,x_{k}^{n_{k}},d_{k}\right)$, and briefly denoted as $x_{k}={\left(x_{k}^{1},x_{k}^{2},\cdots ,x_{k}^{n_{k}}\right)}^{T}$ by removing start node $s_{k}$ and end node $d_{k}$. Let $x={\left(x_{1},x_{2},\cdots ,x_{N_{R}}\right)}^{T}$ denote the matrix whose rows are all paths for all tasks.


## 4. Experiments and Results

### 4.1. Experimental Environment and Parameters

All experiments are conducted on Windows 11 with 16 GB memory, R7-5800H 8 core CPU, 3.2 GHz by using Python 3.7. The experimental tool is PyCharm IDE. Four types of networks are used in the experiments: USANET [10], NSFNET [24], CHNNET [25], and ARPANET [25]. For the details of network topology and other information, please refer to [10,24,25]. The parameters are given in Table 1.

The experiments are divided into four groups. Each group is for one type network and contains four cases according to different numbers of tasks denoted by $N_{R}$ and the number of VNF denoted by $N_{T}$:

Case 1. The number of tasks $N_{R}=50$ and the number of VNF $N_{T}=100$.

Case 2. $N_{R}=100$ and $N_{T}=100$.

Case 3. $N_{R}=150$ and $N_{T}=100$.

Case 4. $N_{R}=200$ and $N_{T}=100$.

To test the stability of the model and algorithms, for each case, the experiments are conducted 10 times, and the mean results are recorded and compared. In the experiments, the population size $N_{pop}=50$ and the maximum generations $N_{gen}=100$.

### 4.2. Comparison of Algorithms 3 and 4

Because the proposed model is a novel one, there is no existing algorithms for the proposed model, we cannot find any suitable existing algorithm as the compared algorithm. We compare the proposed Algorithms 3 and 4 in the experiments.

### 4.3. Performance Measures and Results

In the experiments, we adopt two performance measures: U-measure and C-measure.

1) U-measure [26]:

This metric represents the uniformity and wideness of the Pareto front, which is usually a surface in three-dimensional space. This measure can be calculated by the following three steps:

(1) Determine the boundary of Pareto front.

(2) Determine the nearest neighbors of each Pareto solution in objective space.

(3) Calculate the standard deviation of the distances among the nearest neighbors.

Note that the smaller the U-measure, the better the solution set. For the detail of U-measure, please refer to reference [26].

2) C-measure [27]:

This metric compares the convergent quality of two solution sets. It represents the percentage of solutions in solution set *B* dominated by solutions in solution set *A*. Its definition is as follows:

$$C\left(A,B\right)=\frac{\left|\{u\in B|\exists v\in A:v\;dominates\;u\}\right|}{\left|B\right|}$$

where the numerator represents the number of solutions in *B* dominated by at least one solution in *A*, and the denominator is the number of solutions in *B*.

C(A,B)=1 means all solutions in *B* are dominated by at least one solution in *A* and C(A,B)=0 means no solution in *B* is dominated by solutions in *A*. If C(A,B)>C(B,A), then the solution set *A* has better convergence than solution set *B*.

In the C-measure, for notation convenience, we use *A* to represent the solution set obtained by Algorithm 3 and *B* the solution set obtained by Algorithm 4, respectively. In addition, use $C_{AB}$ and $C_{BA}$ to represent C(A,B) and C(B,A), respectively, where

$$C_{AB}=C\left(A,B\right)=\frac{\left|\{u\in B|\exists v\in A:v\;dominates\;u\}\right|}{\left|B\right|}$$

$$C_{BA}=C\left(B,A\right)=\frac{\left|\{u\in A|\exists v\in B:v\;dominates\;u\}\right|}{\left|A\right|}$$

For each case of one type network, the mean value and standard deviation of each metric in 10 independent runs are recorded. These results are given in Table 2 and Table 3, where each table records the results for one measure on 16 cases of 4 types of networks.

For U-measure, it can be seen from Table 2 that Algorithm 3 generally performs better than Algorithm 4. Among 16 cases of 4 types of networks, Algorithm 3 has the better performance than Algorithm 4 on 12 cases in which Algorithm 3 obtains the smaller values of U-measure. For three cases $\left(N_{T},N_{R}\right)=\left(100,100\right)$, $\left(N_{T},N_{R}\right)=\left(100,150\right)$ and $\left(N_{T},N_{R}\right)=\left(100,200\right)$ of NSFNET, the values of U-measure of Algorithm 3 are 1.0093, 1.0118, and 1.0065, respectively, which are smaller than the values 1.0119, 1.011, and 1.0079 of U-measure of Algorithm 4. For all four cases of USANET, the values of U-measure of Algorithm 3 are 1.0168, 1.0105, 1.0042, and 1.0047, respectively, while the values of U-measure of Algorithm 4 are 1.0181, 1.0119, 1.0077, and 1.0073, respectively. Thus, the values of U-measure of Algorithm 3 are smaller than those of Algorithm 4 for all cases of USANET. Similarly, it can be seen from Table 2 that, for all four cases of ARPANET, the values of U-measure of Algorithm 3 are smaller than those of Algorithm 4, which indicates that Algorithm 3 performs better than Algorithm 4 on U-measure. Only for four cases (one case of NSFNET and three cases of CHNNET) does Algorithm 4 obtain smaller values of U-measure. For the case $\left(N_{T},N_{R}\right)=\left(100,50\right)$ of NSFNET, the value of U-measure of Algorithm 3 is 1.0164 which is larger than that (i.e., 1.0147) of Algorithm 4. For three cases of CHNNET with a smaller number of tasks, the values of U-measure of Algorithm 3 are a little bit larger than the corresponding values of U-measure of Algorithm 4, while for the larger number of tasks (i.e., NR=200), Algorithm 3 outperforms Algorithm 4. Anyhow, both algorithms obtain relatively small values of U-measure on all 16 cases of all types of networks. This illustrates that both algorithms perform well. In addition, note that the standard deviation values for two algorithms are relatively small. This indicates that both algorithms are robust and perform stably.

For C-measure, it can be seen from Table 3 that Algorithm 3 performs completely better than Algorithm 4 with respect to the convergent ability. For all four cases of NSFNET, about 77.27%, 47.25%, 52.6%, and 47.05% solutions obtained by Algorithm 4 are dominated by solutions obtained by Algorithm 3, respectively, while no solution obtained by Algorithm 3 is dominated by solutions obtained by Algorithm 4. For USANET, for case $\left(N_{T},N_{R}\right)=\left(100,50\right)$, 70.39% solutions obtained by Algorithm 4 are dominated by solutions obtained by Algorithm 3, while no solution obtained by Algorithm 3 is dominated by solutions obtained by Algorithm 4. For case $\left(N_{T},N_{R}\right)=\left(100,100\right)$, 69.52% solutions obtained by Algorithm 4 are dominated by solutions obtained by Algorithm 3, while 4.88% solutions obtained by Algorithm 3 are dominated by solutions obtained by Algorithm 4. For case $\left(N_{T},N_{R}\right)=\left(100,150\right)$, 42.16% solutions obtained by Algorithm 4 are dominated by solutions obtained by Algorithm 3, while 9.95% solutions obtained by Algorithm 3 are dominated by solutions obtained by Algorithm 4. For case $\left(N_{T},N_{R}\right)=\left(100,200\right)$, 53.64% solutions obtained by Algorithm 4 are dominated by solutions obtained by Algorithm 3, while 0.83% solutions obtained by Algorithm 3 are dominated by solutions obtained by Algorithm 4. For ARPANET, for case $\left(N_{T},N_{R}\right)=\left(100,50\right)$, 86.9% solutions obtained by Algorithm 4 are dominated by solutions obtained by Algorithm 3, while 2.87% solutions obtained by Algorithm 3 are dominated by solutions obtained by Algorithm 4. For case $\left(N_{T},N_{R}\right)=\left(100,100\right)$, 68.71% solutions obtained by Algorithm 4 are dominated by solutions obtained by Algorithm 3, while 5.0% solutions obtained by Algorithm 3 are dominated by solutions obtained by Algorithm 4. For case $\left(N_{T},N_{R}\right)=\left(100,150\right)$, 58.37% solutions obtained by Algorithm 4 are dominated by solutions obtained by Algorithm 3, while 1.33% solutions obtained by Algorithm 3 are dominated by solutions obtained by Algorithm 4. For case $\left(N_{T},N_{R}\right)=\left(100,200\right)$, 44.62% solutions obtained by Algorithm 4 are dominated by solutions obtained by Algorithm 3, while 2.14% solutions obtained by Algorithm 3 are dominated by solutions obtained by Algorithm 4. For CHNNET, for case $\left(N_{T},N_{R}\right)=\left(100,50\right)$, 93.32% solutions obtained by Algorithm 4 are dominated by solutions obtained by Algorithm 3, while no solution obtained by Algorithm 3 is dominated by solutions obtained by Algorithm 4. For case $\left(N_{T},N_{R}\right)=\left(100,100\right)$, 85.53% solutions obtained by Algorithm 4 are dominated by solutions obtained by Algorithm 3, while no solution obtained by Algorithm 3 is dominated by solutions obtained by Algorithm 4. For case $\left(N_{T},N_{R}\right)=\left(100,150\right)$, 95.36% solutions obtained by Algorithm 4 are dominated by solutions obtained by Algorithm 3, while no solution obtained by Algorithm 3 is dominated by solutions obtained by Algorithm 4. For case $\left(N_{T},N_{R}\right)=\left(100,200\right)$, 97.78% solutions obtained by Algorithm 4 are dominated by solutions obtained by Algorithm 3, while no solution obtained by Algorithm 3 is dominated by solutions obtained by Algorithm 4.

By summary, Algorithm 3 performs better than Algorithm 4 in most cases for all types of networks by using two performance metrics. However, both algorithms can obtain well distributed solution sets.

## 5. Discussion and Future Works

Task scheduling and resource allocation in an elastic optical network is an important and challenging problem. Different providers and customers may have different requirements. With the increase of numbers of tasks and VNF, the problems will become more and more difficult. This is very challenging to design efficient algorithm for the model. However, the proposed multi-objective optimization model in this paper can well satisfy these multiple requirements of both providers and customers as most as possible, and the designed algorithms can solve the model well. In addition, it seems that the proposed algorithm is still effective and efficient with the increase of the numbers of tasks and VNF. However, the limitation of this work is mainly that, when the links of networks are sparse and there are too many tasks, the performance of the proposed model and algorithm will decrease. In addition, the task scheduling and resource allocation in the elastic optical network involves too many issues except for routing, spectrum allocation, VNF deployment, and load balance of data centers. In the future work, it is necessary to study how to set up a new model with more objectives (many objective optimization model) to tackle more issues and satisfy more requirements for service providers and customers in addition to how to design more efficient algorithms for the multi-objective optimization model.

## 6. Conclusions

In this paper, we set up a new multi-objective optimization model for task scheduling and resource allocation problem in elastic optical networks. By using this model, both the total length of paths for all tasks and the totally occupied spectrums can be minimized, which illustrates that the lowest amount of resources are consumed. In addition, the loads on all data centers can be mostly balanced, which illustrates that the efficiency of the network is the highest. Thus, the new model is effective and efficient. To solve the model, we design two new effective evolutionary algorithms. The experiments conducted on up to 16 cases of 4 types of widely used networks have indicated that the proposed algorithms are effective.

## Figures and Tables

**Table 1:** Parameters for four types of networks.

Network Topology	NSFNET	CHNNET	ARPANET	USANET
Number of Nodes	14	15	20	24
Number of links	21	27	32	43
Number of spectrum	358	358	358	358

**Table 2:** Comparison of U-measure.

Networks	($N_{T},N_{R}$)	Algorithm 3		Algorithm 4	
		$U_{\mathrm{mean}}$	$U_{\mathrm{std}}$	$U_{\mathrm{mean}}$	$U_{\mathrm{std}}$
NSFNET	(100,50)	1.0164	0.0073	1.0147	0.0078
	(100,100)	1.0093	0.0030	1.0119	0.0040
	(100,150)	1.0118	0.0060	1.0110	0.0029
	(100,200)	1.0065	0.0032	1.0079	0.0029
USANET	(100,50)	1.0168	0.0072	1.0181	0.0077
	(100,100)	1.0105	0.0089	1.0119	0.0045
	(100,150)	1.0042	0.0011	1.0077	0.0042
	(100,200)	1.0047	0.0024	1.0073	0.0029
ARPANET	(100,50)	1.0134	0.0065	1.0158	0.0064
	(100,100)	1.0078	0.0056	1.0102	0.0052
	(100,150)	1.0069	0.0035	1.0095	0.0043
	(100,200)	1.0059	0.0031	1.0072	0.0018
CHNNET	(100,50)	1.0191	0.0085	1.0151	0.0055
	(100,100)	1.0110	0.0048	1.0091	0.0042
	(100,150)	1.0096	0.0046	1.0080	0.0031
	(100,200)	1.0059	0.0022	1.0068	0.0035

**Table 3:** Comparison of C-measure.

Networks	($N_{T},N_{R}$)	$C_{\mathrm{AB}}$		$C_{\mathrm{BA}}$	
		$C_{\mathrm{mean}}$	$C_{\mathrm{std}}$	$C_{\mathrm{mean}}$	$C_{\mathrm{std}}$
NSFNET	(100,50)	0.7727	0.1449	0	0
	(100,100)	0.4725	0.1760	0	0
	(100,150)	0.5260	0.1671	0	0
	(100,200)	0.4705	0.1131	0	0
USANET	(100,50)	0.7039	0.2410	0	0
	(100,100)	0.6952	0.1279	0.0488	0.0804
	(100,150)	0.4216	0.2087	0.0995	0.0832
	(100,200)	0.5364	0.1452	0.0083	00264
ARPANET	(100,50)	0.8690	0.0872	0.0287	0.0607
	(100,100)	0.6871	0.1356	0.0500	0.1208
	(100,150)	0.5837	0.1544	0.0133	0.0422
	(100,200)	0.4462	0.1484	0.0214	0.0678
CHNNET	(100,50)	0.9332	0.0737	0	0
	(100,100)	0.8553	0.1135	0	0
	(100,150)	0.9536	0.0813	0	0
	(100,200)	0.9778	0.0703	0	0

## Data Availability

For the network framework data, please refer to [18,19,20].

## References

[1] Abkenar F.S., Rahbar A.G. (2017). Study and analysis of routing and spectrum allocation (RSA) and routing, modulation and spectrum allocation (RMSA) algorithms in elastic optical networks (EONs). Opt. Switch. Netw..

[2] Alyatama A. Multi-path routing based on relative cost in elastic optical networks. Proceedings of the 2020 7th International Conference on Electrical and Electronics Engineering (ICEEE).

[3] Zhou Y., Sun Q., Lin S. (2020). Link state aware dynamic routing and spectrum allocation strategy in elastic optical networks. IEEE Access.

[4] Yan B., Zhao Y., Yu X., Wang W., Wu Y., Wang Y., Zhang J. (2018). Tidal-traffic-aware routing and spectrum allocation in elastic optical networks. J. Opt. Commun. Netw..

[5] Al-Yatama A. (2020). Relative cost routing and spectrum allocation in elastic optical networks. J. Opt. Commun. Netw..

[6] Gocień R. (2019). Two metaheuristics for routing and spectrum allocation in cloud-ready survivable elastic optical networks. Swarm Evol. Comput..

[7] Eramo V., Miucci E., Ammar M., Lavacca F.G. (2017). An approach for service function chain routing and virtual function network instance migration in network function virtualization architectures. IEEE/ACM Trans. Netw..

[8] Mechtri M., Ghribi C., Zeghlache D. (2016). A scalable algorithm for the placement of service function chains. IEEE Trans. Netw. Serv. Manag..

[9] Herrera J.G., Botero J.F. (2016). Resource allocation in NFV: A comprehensive survey. IEEE Trans. Netw. Serv. Manag..

[10] Assis K.D.R., Santos A.F., Almeida R.C., Reed M.J., Jaumard B., Simeonidou D. (2020). Virtualization of elastic optical networks and regenerators with traffic grooming. J. Opt. Commun. Netw..

[11] Liu Y., Gu H., Yan F., Calabretta N. (2021). Highly-efficient switch migration for controller load balancing in elastic optical inter-datacenter networks. IEEE J. Sel. Areas Commun..

[12] Mesquita L., Assis K., Pinho L., Almeida R., Filho E., Alencar M. (2021). Resource planning on elastic optical networks using traffic matrix prediction. Int. J. Electron. Commun..

[13] Yin S., Zhang Z., Yang C., Chu Y., Huang S. (2021). Prediction-based end-to-end dynamic network slicing in hybrid elastic fiber-wireless networks. J. Light. Technol..

[14] Xu Y., Zhao Z., Cheng P., Chen Z., Ding M., Vucetic B., Li Y. (2021). Constrained reinforcement learning for resource allocation in network slicing. IEEE Commun. Lett..

[15] Josilo S., Dsn G. (2022). Joint wireless and edge computing resource management with dynamic network slice selection. IEEE-ACM Trans. Netw..

[16] Zhao Y., Zhang Q., Xin X., Li Y., Gao R., Tao Y., Tian Q., Tian F., Chen D., Cao G. (2020). Static resource allocation of advanced reservation requests in elastic optical networks. Appl. Opt..

[17] Miyamura T., Misawa A. (2021). Improving Efficiency of Network Resources in Elastic Optical Transport Network by Using In-Network Cache Functions. Opt. Switch. Netw..

[18] Tang Y., Li X., Gao T., Zhang L., Huang S. (2021). Cost-adaptive multi-class multicast service aggregation based on distributed sub-trees in elastic optical data center networks. Opt. Fiber Technol..

[19] Kitsuwan N., Pavarangkoon P., Nag A. (2020). Elastic optical network with spectrum slicing for fragmented bandwidth allocation. Opt. Switch. Netw..

[20] Khatiri A., Mirjalily G., Luo Z.Q. (2022). Balanced resource allocation for VNF service chain provisioning in inter-datacenter elastic optical networks. Comput. Netw..

[21] Zhu M., Chen Q., Gu J., Gu P. (2022). Deep Reinforcement Learning for Provisioning Virtualized Network Function in Inter-Datacenter Elastic Optical Networks. IEEE Trans. Netw. Serv. Manag..

[22] Kalyanmoy D., Pratap A., Agrawal S. (2002). A fast and elitist multi-objective genetic algorithm: NSGA-II. IEEE Trans. Evol. Comput..

[23] Hershberger J. (2007). Finding the k shortest simple paths: A new algorithm and its implementation. ACM Trans. Algorithms.

[24] Gong L., Zhou X., Liu X., Zhao W., Lu W., Zhu Z. (2013). Efficient resource allocation for all-optical multicasting over spectrum-sliced elastic optical networks. IEEE/OSA J. Opt. Commun. Netw..

[25] Xuan H., Wang Y., Xu Z., Hao S., Wang X. (2017). New bi-level programming model for routing and spectrum assignmentin elastic optical network. Opt. Quantum Electron..

[26] Leung Y.W., Wang Y. (2003). U-measure: A quality measure for multiobjective programming. IEEE Trans. Syst. Man Cybern.

[27] Zitzler E., Deb K., Thiele L. (2000). Comparison of multiobjective evolutionary algorithms: Empirical results. Evol. Comput..

